# Preparation and pharmaceutical properties of Hangeshashinto oral ointment and its safety and efficacy in Syrian hamsters with 5-fluorouracil-induced oral mucositis

**DOI:** 10.1007/s11418-022-01645-y

**Published:** 2022-08-24

**Authors:** Takashi Ogihara, Masato Kagawa, Rintarou Yamanaka, Satoshi Imai, Kotaro Itohara, Daiki Hira, Shunsaku Nakagawa, Atsushi Yonezawa, Michiho Ito, Takayuki Nakagawa, Tomohiro Terada, Kazuo Matsubara

**Affiliations:** 1 Department of Clinical Pharmacology and Therapeutics, Kyoto University Hospital, 54 Shogoin-Kawahara-cho, Sakyo-ku, Kyoto, 606-8507 Japan; 2 Graduate School of Pharmaceutical Sciences, Kyoto University, 46-29 Yoshida-Shimoadachi-cho, Sakyo-ku, Kyoto, 606-8501 Japan; 3 Division of Pharmacognosy, Phytochemistry and Narcotics, Ministry of Health, National Institute of Health Sciences, Labour and Welfare, 3-25-26, Tonomachi, Kawasaki-ku, Kawasaki, Kanagawa 210-9501 Japan; 4 Department of Pharmacy, Wakayama Medical University, Wakayama, 641-8509 Japan

**Keywords:** Chemotherapy-induced oral mucositis, Hangeshashinto, Oral ointment, 5-fluorouracil, Hamster

## Abstract

Chemotherapy-induced oral mucositis (COM) is a common adverse effect of cancer chemotherapy. Several clinical studies reported that repetitive use of mouthwashes containing 2.5–6.25% Hangeshashinto (HST), a Kampo formula, relieves COM, but the effect is insufficient. To solve this problem, we produced an oral ointment of 12% HST extract (considered quantitatively equivalent to 20% commercially available HST), which will increase the local concentrations of its active ingredients and prolong the contact time with COM. In this study, we evaluated the pharmaceutical properties (spreadability and stability) of HST oral ointment. In addition, its safety (oral mucosal irritation) and therapeutic effects on 5-fluorouracil-induced oral mucositis were evaluated in male Syrian hamsters. The HST ointment showed good spreadability and stability for more than 8 weeks at 4 °C. In the oral mucosal irritation test, topical application of HST ointment (0.2 g) three times per day for 14 days had no adverse effect on the oral mucosa of hamsters. In hamsters treated with 5-fluorouracil (60 mg/kg) twice, COM was induced by a submucosal injection of 5% acetic acid into the cheek pouch. When HST ointment (50 µg) was topically applied to the mucositis area once per day for 12 days, the area and macroscopic score of mucositis were significantly decreased, and the depth of the wound tended to be reduced compared with the lactose ointment-treated control animals. These findings suggest that HST oral ointment shows good properties in spreadability, stability, and safety, and elicits a therapeutic effect in an animal model of COM.

**Graphical abstract:**

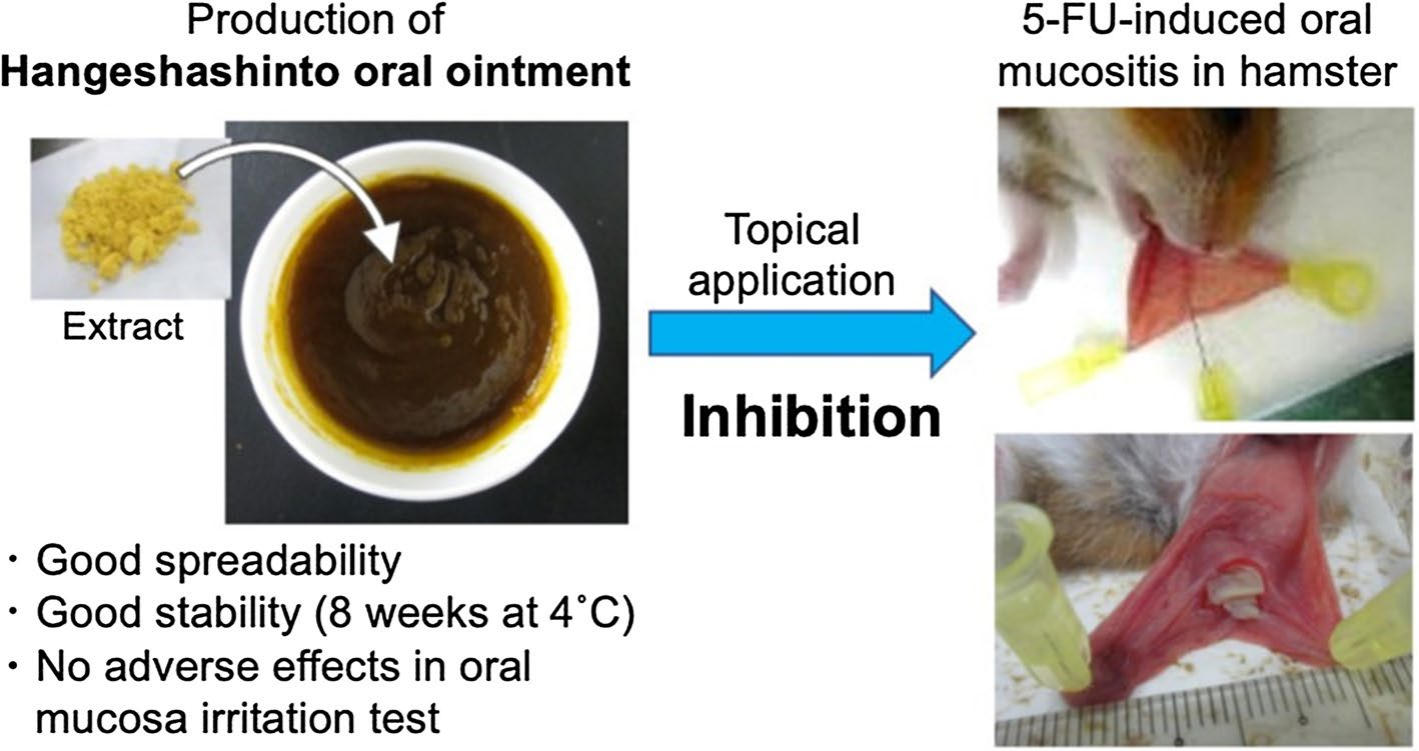

**Supplementary Information:**

The online version contains supplementary material available at 10.1007/s11418-022-01645-y.

## Introduction

Oral mucositis occurs frequently during cancer treatment, with a frequency of approximately 20–40% upon conventional chemotherapy, 70–90% among patients receiving high-dose chemotherapy as conditioning for hematopoietic stem cell transplantation, and higher than 90% upon chemoradiation therapy of head and neck cancers.^1–4)^ Among anticancer drugs, the incidence of oral mucositis is particularly high upon treatment with cytotoxic anticancer drugs such as alkylating drugs, antimetabolites, and platinum-based agents [1–4]. Chemotherapy-induced oral mucositis (COM) causes erythematous ulcerative lesions, oral pain, bleeding, difficulties in eating, mouth opening, chewing, and swallowing, and associated dysgeusia, resulting in a decrease in the patient’s quality of life. In addition, severe COM reduces food intake, leading to a poor nutritional status and dehydration, and increase the risk of infections, which may result in dose reduction or discontinuation of cancer treatment [5]. To prevent COM, basic oral care, including oral hygiene management and mouthwashes, is generally used. To treat oral mucositis pain, mouthwashes containing topical anesthetics and systemic analgesics including acetaminophen and nonsteroidal anti-inflammatory drugs are used, although many patients require opioid analgesics when COM is severe [6, 7]. However, there is no standard prophylactic and therapeutic pharmacotherapy for COM.

Hangeshashinto (HST), a Kampo formula, consists of seven crude drugs: *Pinelliae Tuber*, *Scutellariae Radix*, *Glycyrrhizae Radix*, *Zizyphi Fructus*, *Ginseng Radix*, *Zingiberis Processum Rhizoma*, and *Coptidis Rhizoma* (Table 1). Although HST is empirically used, it is approved for treatment of gastrointestinal diseases and symptoms including dyspepsia, nervous gastritis, gastrasthenia, and oral mucositis in Japan. Accumulating evidence suggests that HST has a variety of pharmacological properties such as anti-inflammatory [8, 9], analgesic [10, 11], antioxidant [12], broad-spectrum antibacterial [13, 14] and wound-healing effects [15] in the oral cavity. These effects can help to alleviate oral ulcerative mucositis including COM. In animal model, it is reported that HST alleviated 5-fluorouracil (5-FU)-induced COM [14, 15]. Furthermore, several clinical studies showed that repetitive use of mouthwashes with 5% HST solution (as a medical product, TSUMURA Hangeshashinto Extract Granules, TJ-14) reduces the duration of COM in patients with gastric and colorectal cancers [16–18]. Similarly, mouthwashes with 2.5% or 6.25% TJ-14 solution improve the rate of chemoradiation completion and the nutritional status of patients with head and neck cancer [19–21]. However, these effects are insufficient to prevent COM because the treatment did not affect the incidence of COM, which was set as a primary endpoint, in these clinical studies. Furthermore, TJ-14-containing mouthwashes do not change the duration of severe COM [17]. This may be because the contact time of the active ingredients of HST with oral mucositis is limited, and the concentrations of active ingredients are lower than required for local treatment.

**Table 1:** Herbal medicines comprising Hangeshashinto and their daily doses [22]

Latin name	g/daily dose
*Pinelliae Tuber*	5.0
*Scutellariae Radix*	2.5
*Glycyrrhizae Radix*	2.5
*Zizyphi Fructus*	2.5
*Ginseng Radix*	2.5
*Zingiberis Rhizoma Processum*	2.5
*Coptidis Rhizoma*	1.0

Oral ointment formulations are effective means to deliver a drug locally in the oral cavity for longer than mouthwashes. In this study, we produced an oral ointment of 12% HST extract, which is considered quantitatively equivalent to 20% TJ-14, and examined its pharmaceutical properties such as spreadability and stability. Furthermore, the safety and efficacy of HST oral ointment were determined in Syrian hamsters.

## Materials and methods

### Preparation of 12% (w/w) HST and 20% (w/w) TJ-14 oral ointments

Seven herbal medicines including *Pinelliae Tuber* (Lot No. 15079G169), *Scutellariae Radix* (Lot No. 18036C296), *Glycyrrhizae Radix* (Lot No. 29054F024), *Zizyphi Fructus* (Lot No. 11116A247), *Ginseng Radix* (Lot No. 04076G056), *Zingiberis Rhizoma Processum* (Lot No. 13115K165), and *Coptidis Rhizoma* (Lot No. 04115K055), which conformed to the Japanese Pharmacopiea 18th Edition [22], were purchased from Nakajima Syoyaku Co. (Kyoto, Japan). Voucher specimens had been deposited in our laboratory for future reference, but we cannot deposit them in a publicly available herbarium. The seven herbal medicines (18.5 g) were mixed according to their required daily doses of HST (Table 1) [22], and boiled for 60 min with 600 mL water in an electric decoction glass pot, which are prescribed for the treatment of several diseases under the oversight of the Japanese Ministry of Health, Labour and Welfare. The decoction was filtrated with Miracloth, while it was hot and then freeze dried with a 28.7% yield (5.31 g dry extract was obtained from 18.5 g mixed herbal medicines). To prepare 10 g ointment, 1.2 g (12%) of the resulting extract was mixed with 2.0 g (20%) sodium carboxymethylcellulose (CMC-Na; Maruishi Pharmaceutical Co. Ltd., Osaka, Japan) that had been passed through a 200 mesh sieve, 2.0 g (20%) liquid paraffin (Maruishi Pharmaceutical Co. Ltd), and 4.8 g (48%) Plastibase (Taisho Pharma Co., Ltd., Tokyo, Japan). The 12% (w/w) HST oral ointment was prepared by mixing three times at 1000 rpm for 30 sec using an automatic ointment mixer (THINKY Co., Tokyo, Japan) and stored at 4 °C until use. Control oral ointment was prepared by the same method using 12% (w/w) lactose instead of 12% (w/w) HST extract.

TSUMURA Hangeshashinto Extract Granules (TJ-14; Lot No. M41982) was manufactured by Tsumura Co. (Tokyo, Japan) according to Japanese and International manufacturing guidelines. TJ-14 contain 40% lactose. Some experiments used 20% (w/w) TJ-14 oral ointment, which is considered quantitatively equivalent to 12% (w/w) HST oral ointment. To prepare 10 g ointment, 2.0 g (20%) TJ-14 was mixed with 2.0 g (20%) CMC-Na, 2.0 g (20%) liquid paraffin, and 4.0 g (40%) Plastibase, as described above. Equivalence between 12% HST ointment and 20% TJ-14 ointment has not been verified.

### Spreadability test

The spreadability of the oral ointment was measured with a spreadmeter (Imoto Machinery Co. Ltd., Kyoto, Japan) according to Supplement II of the Japanese Pharmacopoeia 18th Edition [22]. Half a milliliter of ointment was set in the central cavity of the spreadmeter. The piston was pushed up to eject the specimen onto the lower plate, and then the upper plate (115 g) fell vertically onto the ointment. When the ointment spread for 60 sec forming a circle, the diameter was measured.

### Stability test

Freshly prepared 12% HST ointment samples were stored at 4 °C or 25 °C for 14, 28, and 56 days. Thereafter, the changes in the amounts of 4 marker ingredients (baicalin, glycyrrhizin, berberine, and [6]-shogaol) extracted from the ointment were determined by high-performance liquid chromatography (HPLC). Ingredients were extracted from the ointment using a previously published protocol with slight modifications [23]. One hundred milligrams of the ointment was weighed and placed in a glass centrifuge tube (7 mL). Three milliliters of hexane saturated with methanol was added and mixed vigorously. Then, 2 mL methanol saturated with hexane was added and shaken vigorously, and the tube was centrifuged at 1500 rpm for 5 min at 4 °C. The upper layer was removed, and 3 mL fresh hexane saturated with methanol was added. The extraction was performed three times. After the upper layer was discarded, the methanol layer and precipitate were transferred to a small vial, and the solvent was evaporated using a rotatory evaporator. Residual compounds were dissolved in 2 mL methanol and sonicated for 30 min. The mixture was transferred to a 2 mL centrifuge tube and centrifuged at 1500 × g for 5 min. The supernatant was filtered through a syringe filter (pore size = 0.45 µm), and 20 µL of the filtrate was analyzed by HPLC.

The HPLC apparatus consisted of the following components: an LC-20AD pump, an SIL-20AC HT autosampler, a CTO-20AC column oven, and an SPD-20AV UV detector (Shimadzu, Kyoto, Japan). Baicalin, glycyrrhizin, and berberine were analyzed according to the protocol of Okamura et al. [24] with slight modifications. Two mobile phases were used: mobile phase A was water, and mobile phase B was a mixture of water and acetonitrile at a 20:80 ratio, both containing 0.2% SDS and 200 µL/L 85% phosphoric acid. The gradient program was as follows: from 24% to 28% B in 10 min, from 28% to 50% B in 4 min, from 50% to 54% B in 3 min, from 54% to 67% B in 13 min, and 67% B for 10 min. A re-equilibration period of 20 min was set between each run. Separation was performed with a CHEMCOSORB 5-ODS-H reversed phase column (5 µm particle size; 150×4.6 mm i.d.; Chemco Plus Scientific, Osaka, Japan). The flow rate was 1.0 mL/min, and the column oven temperature was 45℃. The detection wavelength was 265 nm. [6]-Shogaol was analyzed according to the protocol of Kano et al. [25] with slight modifications. The mobile phase was a mixture of acetonitrile, water, and tetrahydrofuran at a ratio of 48:52:0.5. Separation was performed with a TSKgel ODS-120T reversed phase column (5 µm particle size; 250×4.6 mm i.d.; Tosoh, Tokyo, Japan). The flow rate was 1.2 mL/min and the column oven temperature was 45 °C. The detection wavelength was 240 nm. The acquired data were analyzed with the computer program LC Solution (Shimadzu). The value of each ingredient extracted from the ointment was expressed as a percentage of the value on the preparation day.

### Animals

The animal experimental procedures were performed according to the ethical guidelines of the Kyoto University Animal Research Committee (Approval No. 17-58) or the Drug Safety Testing Center (Approval No. IACUC100645RR) and the basic guidelines for the conduct of animal experiments in implementing agencies under the jurisdiction of the Ministry of Health, Labour and Welfare of Japan. The study was performed in a manner of reduced the animal numbers and minimized animal suffering. Five-week-old male golden Syrian hamsters initially weighting 70–100 g were purchased from Japan SLC (Shizuoka, Japan). Hamsters were individually kept in a plastic cage under controlled temperature (22 ± 3.0 °C) and humidity (55 ± 20%) with alternate light-dark cycles from 8:00 a.m. to 8:00 p.m. and given free access to water and food.

### Oral mucosa irritation test in hamsters

The oral mucosa irritation test was performed as a pre-clinical safety testing for 20% TJ-14 oral ointment according to the “ISO 10993-10, Third edition 2010-08-01, Biological evaluation of medical devices-Part 10: Tests for Irritation and Skin Sensitization, Annex B.3 Oral mucosa irritation test” and similar guidelines issued in Japan. Six hamsters were randomly divided into 2 groups (3 hamsters per group): the sham-operated group and TJ-14 ointment-treated group. Under isoflurane anesthesia, the left and right cheek pouches of the hamster were everted and washed with physiological saline. Then, 20% TJ-14 oral ointment (0.2 g) was applied to the right cheek pouch using a Teflon rod, while the left cheek pouch was untreated. The pouches were carefully put back into its original place. One hour after the treatment, the cheek pouches were washed with physiological saline, and the applied ointment was removed with a paper towel. These processes were repeated three times per day for 14 days. Sham-operated animals received the same treatment except for ointment application.

The animals were weighed on days 1, 8, and 14, and the general and macroscopic statuses of both cheek pouches were observed after each treatment and 24 h after the last treatment. The appearance of the cheek pouches was recorded, and the pouch surface reactions were graded macroscopically for erythema on a scale of 0 to 4 (Supplementary Table 1).

For histological examination, 24 h after the last treatment and following macroscopic observation, animals were sacrificed by exsanguination under anesthesia with sodium pentobarbital. The treated cheek pouches were excised and fixed in 10% neutral buffered formalin. Following fixation, the specimens were dehydrated in an ethanol series, embedded in paraffin, sectioned, and stained with hematoxylin and eosin (HE) for microscopy. The irritant effects on the oral mucosa in the distal, middle, and proximal sites of the cheek pouch were graded microscopically for epithelium, leucocyte infiltration, vascular congestion, and edema on a scale of 0 to 4 (Supplementary Table 1). The histological scores for each site were summed. Irritation was graded as follows according to the total score: 0, none; 1–4, minimal; 5–8, mild; 9–11, moderate; and 12–16, severe.

### 5-FU-induced COM hamster model and drug administration

Oral mucositis was induced according to the protocol of Yoshino et al. [26] with slight modifications. Forty-six hamsters were randomly divided into 3 groups: no treatment control group (6 hamsters), lactose ointment-treated group (8 hamsters for macroscopic analysis and 6 hamsters for histological assessment), HST ointment-treated group (8 hamsters for macroscopic analysis and 6 hamsters for histological assessment), 5% TJ-14 solution-treated group (6 hamsters) and water-treated group (6 hamsters). The hamster was intraperitoneally administered 60 mg/kg 5-FU on days -2 and 0. On day 0, under isoflurane anesthesia, the left cheek pouch of the hamster was everted and fixed to expanded polystyrene with 30G needles. Twenty microliters of 5% acetic acid was injected under the cheek pouch mucosa using a microsyringe and a 30G needle to induce mucositis, and then the hamster was returned to the home cage. To eliminate the effects of excipients in TJ-14 on COM, 12% HST oral ointment was used. Under isoflurane anesthesia, 12% HST or lactose oral ointment (50 μg) was applied using a cotton swab to the mucositis area once per day from day 1 to day 12. In the no treatment control group, the animals were not treated with any ointment. To mimic clinical studies by mouthwashes with TJ-14 solution [16–21], 5% TJ-14 solution or water (200 μL) was applied using a syringe in the left cheek pouch twice per day from day 1 to day 12. Hence, in the groups treated with 12% HST oral ointment (50 μg) and 5% TJ-14 solution (200 μL), the same amount of HST extract (6 μg) was applied to the mucositis area, although the former was less frequent (once per day) that the later (twice per day) for the same period.

The animals were included, if the macroscopic score of oral mucositis was greater than or equal to 2 on day 1. The animals were excluded if the mucositis score was 1 or less on day 1, or if the animal died during the observation period, preventing the collection of behavioral and histological data. However, no animals were excluded in the present study.

### Macroscopic analyses of oral mucositis

The primary outcome of this study will be macroscopic assessments of COM. The cheek pouch mucosa was photographed from day 1 (just before applying the ointment) to day 12 for measurement of the mucositis area and macroscopic scoring of oral mucositis. The area of mucositis was measured using the free computer program ImageJ (National Institute of Health, Bethesda, MD, USA). The area on each day was expressed as a percentage of the area on the first day. The macroscopic score was graded by the same trained experimenter based on the criteria of Lima et al. [27] as shown in Table 2.

**Table 2:** Grade for macroscopic oral mucositis of hamster cheek pouches^25)^

Grade 0	Normal cheek pouch with absent or discreet erythema and hyperemia with no hemorrhagic areas, ulcerations, or abscesses
Grade 1	Moderate erythema and hyperemia with no hemorrhagic areas, ulcerations, or abscesses
Grade 2	Severe erythema and hyperemia with hemorrhagic areas, small ulcerations, or scarred tissue, but no abscesses
Grade 3	Severe erythema and hyperemia with hemorrhagic areas, extensive ulcerations, and abscesses

### Histological examination

The secondary outcome of this study will be histological assessments of COM. 5-FU-induced COM hamsters treated with lactose or HST ointment were deeply anesthetized on day 7 with sodium pentobarbital (50 mg/kg, i.p.; Nacalai Tesque) and transcardially perfused with potassium-free phosphate-buffered saline followed by 4% paraformaldehyde prepared in 0.1 M phosphate buffer. Subsequently, the left cheek pouches of hamsters were quickly harvested and post-fixed overnight in 4% paraformaldehyde. The specimens were then dehydrated, embedded in paraffin, and sliced at a thickness of 10 µm. The obtained sections were subjected to HE staining and then observed by microscopy. The depth of the wound was calculated using ImageJ. The sections were excluded from data analyses because of technical errors in the histological analysis procedure.

### Statistical analysis

Data were analyzed using GraphPad Prism version 8 (GraphPad Software, San Diego, CA, USA) and are presented as means ± standard error of the mean (S.E.M.). Data were compared between two groups using the Student’s t test. Data were compared between more than two groups using a one-way or two-way analysis of variance (ANOVA) followed by Bonferroni’s post hoc test. Time course data were analyzed using a two-way ANOVA for repeated measures followed by Bonferroni’s post hoc test. In all cases, *P* < 0.05 was considered statistically significant.

## Results

### Spreadability and stability of HST and TJ-14 oral ointments

**Fig. 1:**
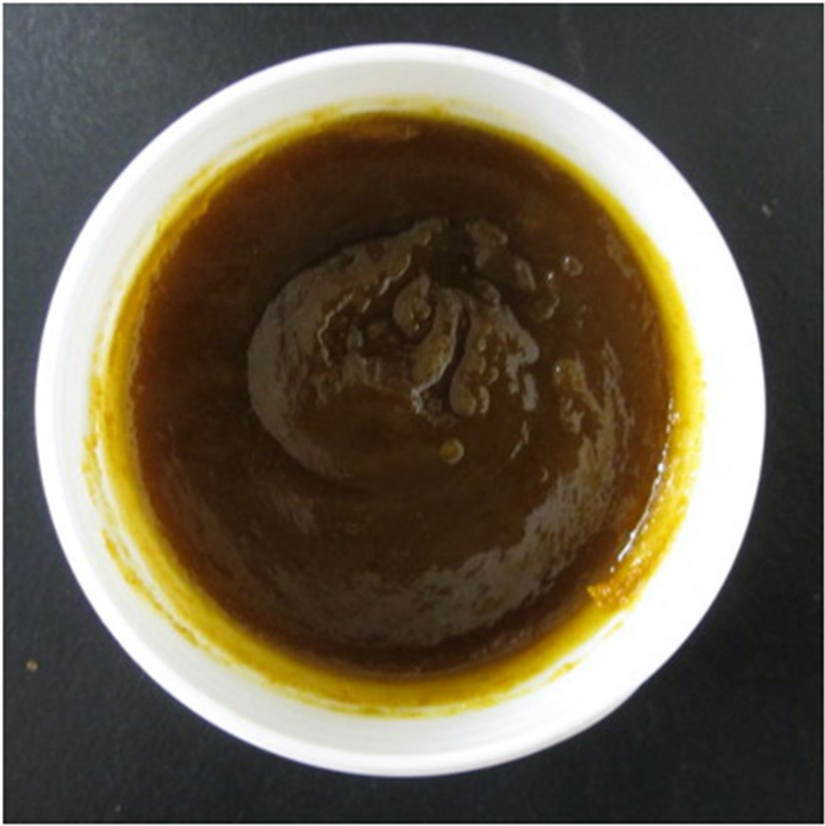
Appearance of 12% HST oral ointment

Figure 1 shows the appearance of 12% HST oral ointment. In the spreadability test, the diameters of 12% HST oral ointment and 20% TJ-14 oral ointment were 3.0 and 2.6 cm, respectively.

**Fig. 2:**
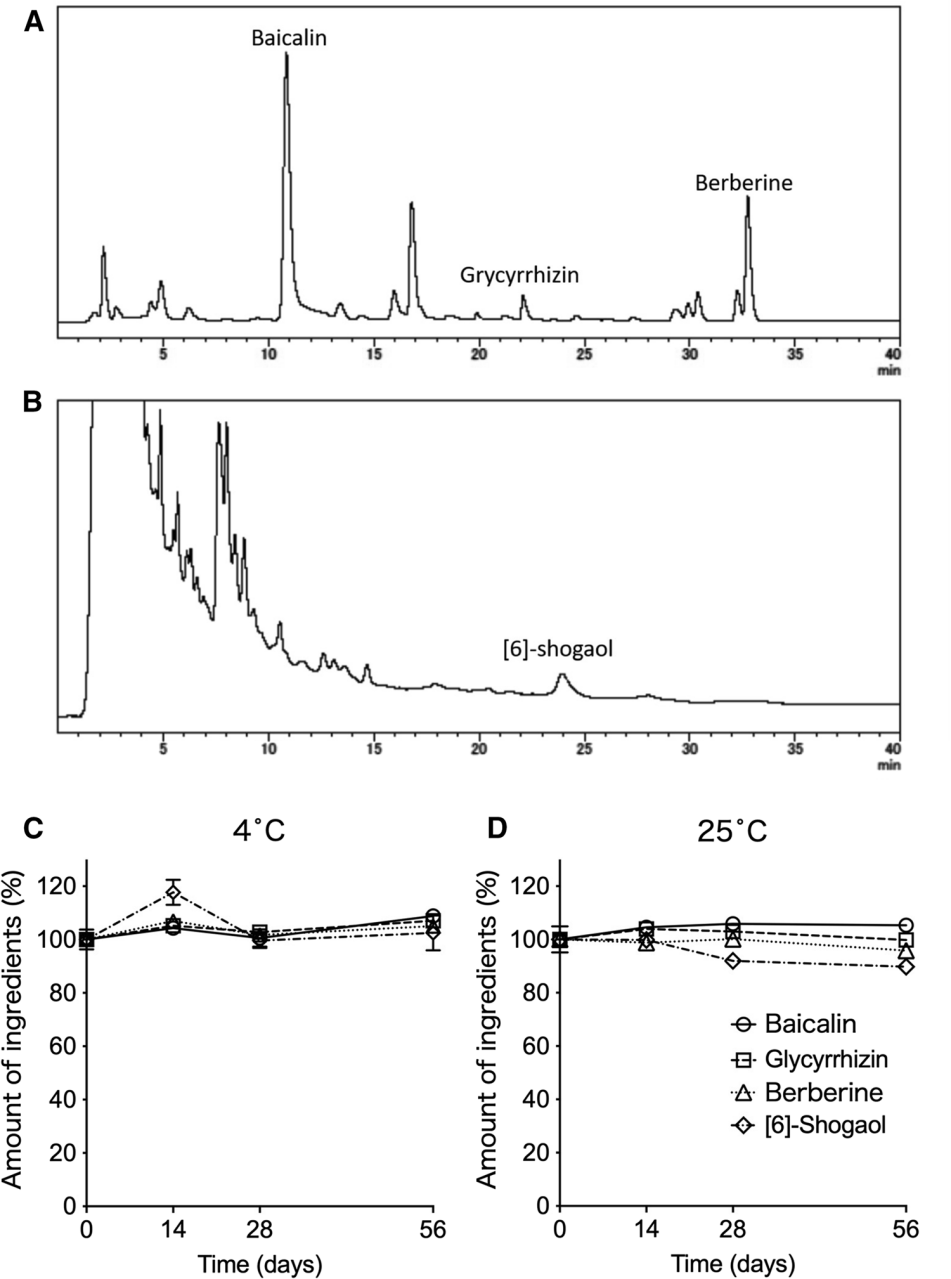
Stability test of 12% HST oral ointment. The major ingredients extracted from 12% HST oral ointment were analyzed by HPLC. **A** A representative HPLC chromatogram generated according to the protocol of Okamura et al.^5)^ The retention times of baicalin, glycyrrhizin, and berberine were 10.9, 22.1, and 32.8 min, respectively. **B** A representative chromatogram generated according to the protocol of Kano et al.^6)^ The retention time of [6]-shogaol was 23.9 min. **C**, **D** Changes in the amounts of marker ingredients extracted from 12% HST oral ointment stored at 4 °(C) or 25°°C (D) for 14, 28, and 56 days. The value of each ingredient on each day is expressed as a percentage of the value on day 0. Each point represents mean ± SEM. *n* = 4.

Figures 2A and 2B show representative HPLC chromatograms of 12% HST oral ointment. Figures 2C and 2D and Supplementary Figure 1 show the changes in the amounts of four marker ingredients (baicalin, glycyrrhizin, berberine, and [6]-shogaol) extracted from the ointment stored for 14–56 days at 4 °C and 25 °C, respectively. The amounts of these ingredients were not reduced throughout the experimental period when the ointment was stored at 4 °C. However, the mean amount of [6]-shogaol gradually decreased when the ointment was stored at 25 °C. No significant change in the appearance, including color, of HST ointment was observed (data not shown).

### Oral mucosa irritation of TJ-14 oral ointment

**Fig. 3:**
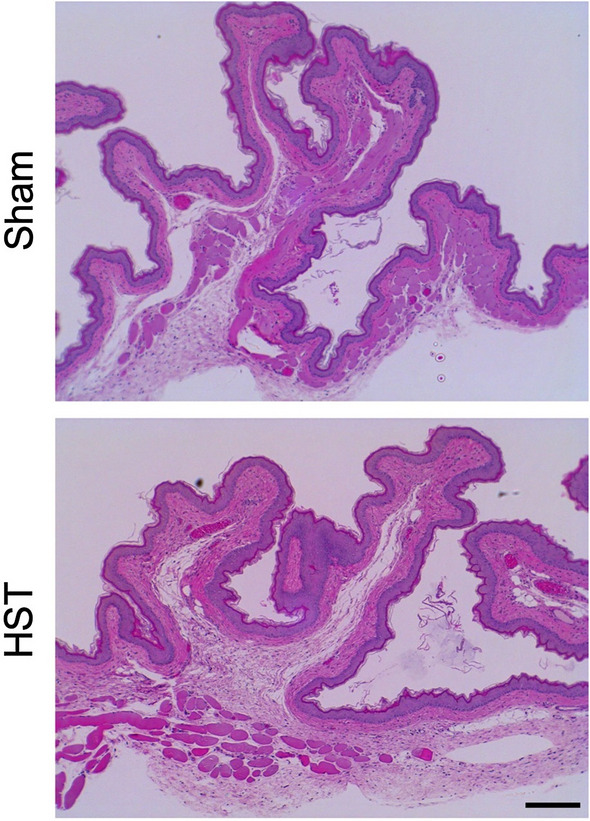
Histological analysis of the oral mucosa of hamsters after HST oral ointment application in the oral mucosa irritation test. In this experiment, 20% TJ-14 oral ointment (0.2 g) was applied to the cheek pouch of hamsters three times per day for 14 days. Control (sham) animals received the same treatment except for ointment application. No significant changes were observed in the sham-operated or TJ-14 ointment-treated group. Scale bar = 200 μm.

In this experiment, 20% TJ-14 oral ointment (0.2 g) was applied to the cheek pouch of hamsters three times per day for 14 days. Control animals received the same treatment except for ointment application. Application of TJ-14 ointment did not cause any abnormal changes in the general status of the animals or change their body weight (Table 3). No animals in the sham-operated or TJ-14 ointment-treated group exhibited erythema or crusting of the mucosal surface of the right or left cheek pouch (data not shown). The macroscopic score for irritation was 0 in all cases throughout the treatment period (Supplementary Table 2). In histological examinations, no significant changes were observed in the sham-operated or TJ-14 ointment-treated group (Fig. 3). In both groups, the mean irritation scores for the epithelium, leucocyte infiltration, vascular congestion, and edema were 0 in the distal, middle, and proximal regions (Supplementary Table 3).

**Table 3:** Effect of topical application of TJ-14 oral ointment on body weight (g) of hamsters

	Sham	TJ-14 ointment
Day 1	103.4 ± 2.7	103.6 ± 3.0
Day 8	105.0 ± 5.4	106.0 ± 7.9
Day 14	109.9 ± 7.4	112.6 ± 10.3

### Effects of HST oral ointment on COM in hamsters

**Fig. 4:**
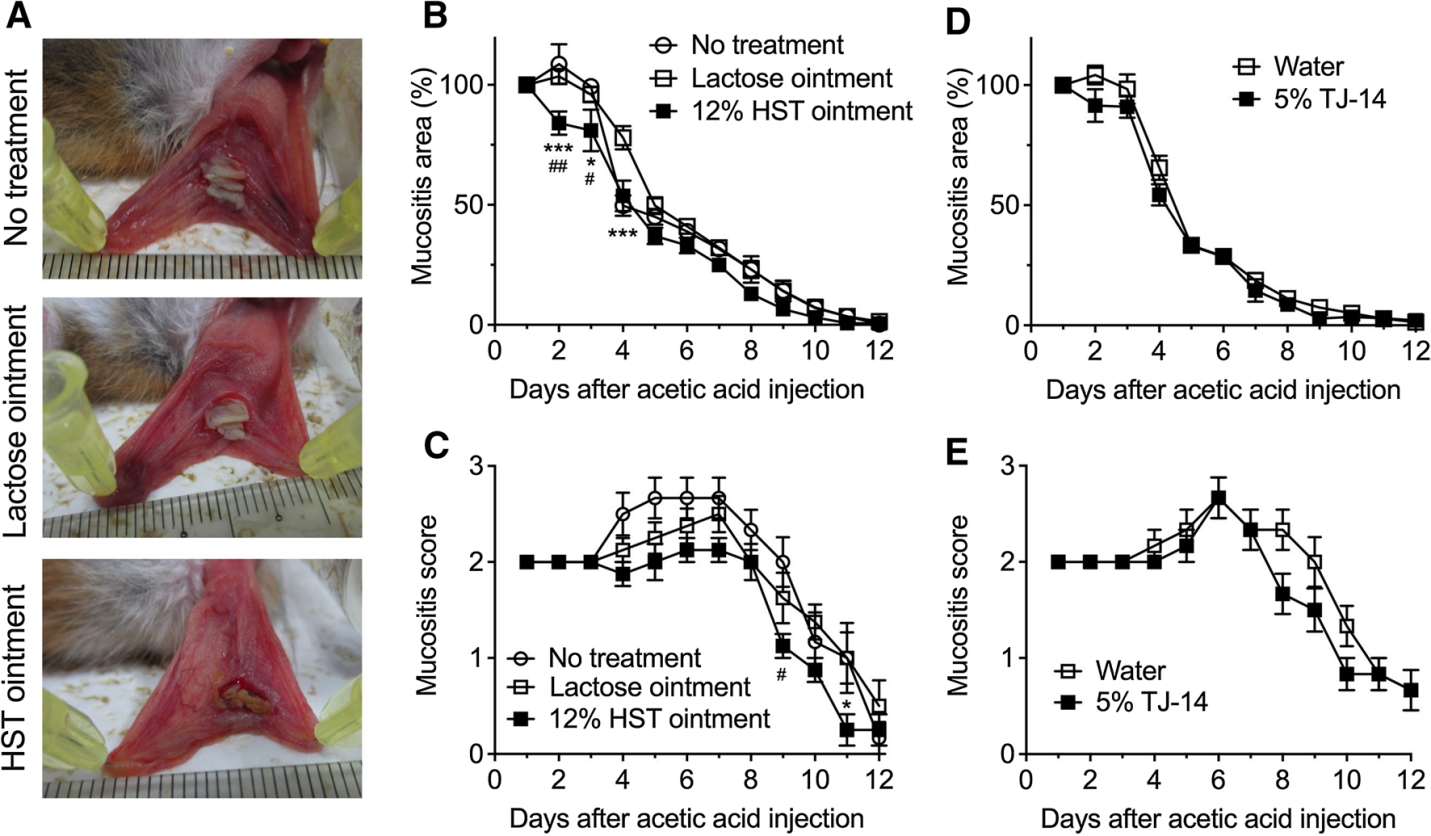
Effects of topical application of 12% HST oral ointment on the area and score of oral mucositis in hamsters with 5-FU-induced COM. Hamsters were given 5-FU (60 mg/kg, i.p.) on days –2 and 0. To evoke oral mucositis, 5% acetic acid (20 μL) was injected under the cheek pouch mucosa on day 0. (**A**–**C**) 12% HST or lactose oral ointment (50 μg) was applied to the mucositis area once per day from day 1 to day 12. In the no treatment control group, animals were not treated with any ointment. (A) Appearance of cheek pouches on day 7. (B) Change in the area of oral mucositis of no treatment control group (*n* = 6), lactose ointment-treated group (*n* = 8) and HST ointment-treated group (*n* = 8). The area on each day is expressed as a percentage of the area on the first day. (C) Change in the macroscopic score of oral mucositis. (D–E) 5% TJ-14 solution or water (200 μL) was applied in the left cheek pouch twice per day from day 1 to day 12. (D) Change in the area (%) and macroscopic score of oral mucositis of water-treated (*n* = 6) and TJ-14 solution-treated groups (*n* = 6). Each point represents mean ± SEM. ^#^*P* < 0.05, ^##^*P* < 0.01 vs. no treatment group; *P < 0.05, ***P < 0.001 vs. lactose oral ointment-treated group (repeated two-way ANOVA followed by Bonferroni’s post hoc test).

In hamsters treated with 5-FU (60 mg/kg) twice, a submucosal injection of 5% acetic acid at day 0 induced oral mucositis. In hamsters with COM, 12% HST or lactose oral ointment (50 μg) was applied once per day from day 1 to day 12. Figure 4A shows photographs of the cheek pouches of hamsters with COM on day 7. The effects of lactose and HST ointment on the area and macroscopic score of oral mucositis in the COM hamster model were examined. In the no treatment control and lactose ointment-treated groups, the area of oral mucositis rapidly expanded at 1 day after acetic acid injection and then gradually decreased and disappeared within 12 days after the injection. The macroscopic score of oral mucositis gradually increased over 5–7 days and then decreased and reached 0 within 12 days after the injection. Topical application of HST ointment significantly reduced the area of oral mucositis (*F*_2,19_ = 8.19, *P* = 0.0027). The area of oral mucositis significantly differed between the HST ointment-treated group and the no treatment control and lactose ointment-treated groups at 2, 3, and 4 days after the acetic acid injection (Fig. 4B). The macroscopic score of oral mucositis was also lower in the HST ointment-treated group than in the no treatment control and lactose ointment-treated groups throughout the observation period (*F*_2,19_ = 3.33, *P* = 0.0578), and this difference was significant at 9 and 11 days after the injection (Fig. 4C). By contrast, topical application of 5% TJ-14 solution in the cheek pouch twice per day had no significant effects on the area (*F*_1,10_ = 2.36, *P* = 0.208) and the macroscopic score (*F*_1,10_ = 2.37, *P* = 0.155) of oral mucositis, compared with water-treated group, although it tended to reduce the macroscopic score at the recovery phase (Fig. 4D and 4E).

**Fig. 5:**
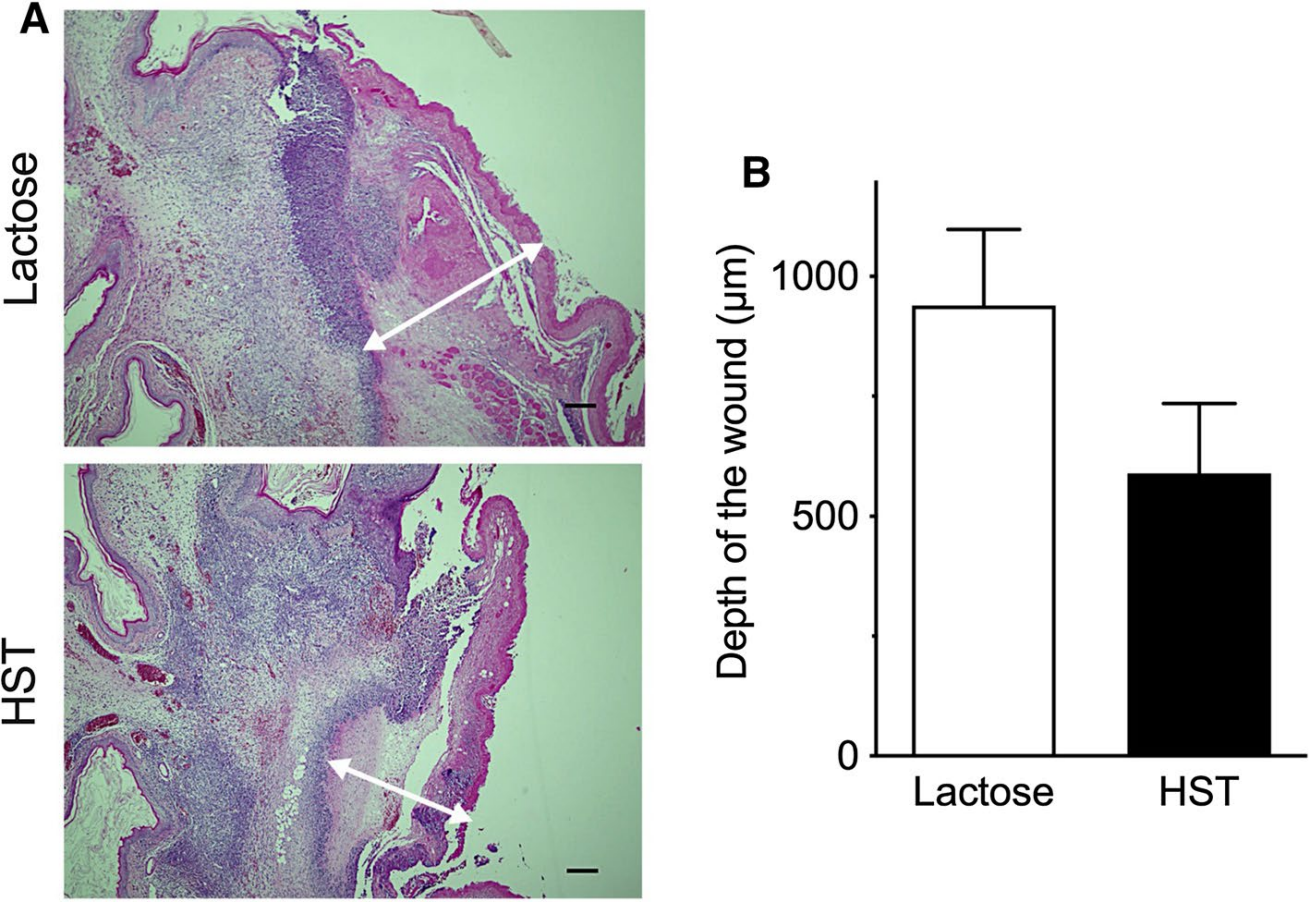
Histopathological analysis of oral mucositis in hamsters with 5-FU-induced COM following topical application of 12% HST or lactose oral ointment. **A** Representative photomicrographs of oral mucositis on day 7. Double-headed arrows indicate the depth of the mucosal wound. Scale bar = 200 μm. **B** Depth of the mucosal wound of lactose ointment-treated group (*n* = 6) and HST ointment-treated group (*n* = 6). There was no significant difference between the groups. Each bar represents mean ± SEM.

Figure 5A shows histopathological photomicrographs of the oral mucositis stained with HE on day 7. Application of HST ointment reduced the depth of the mucosal wound. The mean depths of the mucosal wound were 939.0 ± 159.0 µm in the lactose ointment-treated group and 590.1 ± 144.6 µm in the HST ointment-treated group, respectively. However, this difference was not statistically significant (Fig. 5B).

## Discussion

In this study, we prepared an oral ointment containing 12% HST extract, as well as 20% TJ-14 oral ointment, and showed its good properties in spreadability and stability. Topical applications of the ointment for a relatively long period had no adverse effects in the oral mucosa irritation test. Furthermore, it inhibited oral mucositis in the 5-FU-induced COM hamster model, rather than topical application of TJ-14 solution. These findings suggest that HST oral ointment is safe and efficacious to prevent or treat COM.

The HST oral ointment contains 20% CMC-Na, which absorbs saliva to form a gel and helps the ointment stay in the oral cavity. Thus, the ointment is considered to exert longer-lasting effects on oral mucositis than mouthwashes containing HST. In addition, the HST oral ointment contains 12% HST extract or 20% TJ-14, while HST mouthwashes used in previous clinical studies only contain 2.5–6.25% TJ-14. [16–20] However, this preparation must have appropriate pharmaceutical properties for use as an oral ointment. Spreadability is an important property of oral ointments because it affects the ease of their application, especially in the oral cavity. The spreadabilities of 12% HST and 20% TJ-14 oral ointments (3.0–2.6 cm, respectively) are in the appropriate range for clinically used skin ointments (about 2.0–3.5 cm). The commercially available oral ointments Kenalog^®^ (Bristol-Myers Squibb, Tokyo, Japan) and Dexaltin^®^ (Nippon Kayaku, Tokyo, Japan) spread to 2.6 and 2.8 cm in 60 sec, respectively, based on a previous study [28]. These data suggest that the viscosities of 12% HST and 20% TJ-14 oral ointments are appropriate for clinical use.

In the stability test, the HST ointment was stable for at least 8 weeks when stored at 4 °C. When stored at 25 °C, the amount of [6]-shogaol extracted from the ointment slightly decreased, although the amounts of other marker ingredients did not change. Thus, it is desirable to store the HST ointment at 4 °C. In clinical settings, COM develops soon after chemotherapy initiation, peaks in 7–10 days, and heals within 2 weeks [29]. Stability of the ointment for 8 weeks is sufficient to cover the duration of COM.

Some ingredients of HST are irritative, and therefore the local adverse effects on the oral mucosa may be pronounced using HST ointment with higher concentration and a longer contact time than HST-containing mouthwash. However, in the oral mucosal irritation test, no adverse effect was detected even with a larger volume and longer application of 20% TJ-14 oral ointment, demonstrating its safety, in the oral mucosa at least. In addition, it did not affect the general status or body weight of hamsters. The dosage for topical 20% TJ-14 oral ointment is low at less than one-tenth of that for oral administration of TJ-14 granules; therefore, systemic adverse effects of HST oral ointment are not considered a problem.

The COM hamster model induced by submucosal injection of acetic acid into the cheek pouch of 5-FU-treated hamsters has been frequently used to assess the effects of drugs on COM [26, 30, 31], and to obtain pharmacological data in nonclinical test for the approval of oral mucositis drugs. Mucositis was evaluated by measuring the area of the erosive and ulcerative region and calculating the scores based on multiple findings such as erythema, bleeding, hyperemia, and abscesses in addition to erosion and ulcers. In the present COM hamster model, an erosion developed in the early stages of mucositis. This was followed by hyperemia and abscess exacerbation and ulceration in about 7 days, while the erosive and ulcerative area gradually shrank. These features could be responsible for the difference in the time courses of the area and score. Overall, HST oral ointment attenuated the area and score of COM throughout the observation period. Although the effects were only significant on particular days, the degree of inhibition is similar to that reported previous studies using other drugs [26, 30, 31]. Consistent with the present results, several studies reported that HST inhibits oral mucositis in animal models. Continuous oral administration of TJ-14 (diet containing 2% TJ-14) attenuates the mucositis score and neutrophil infiltration in hamsters with radiation-induced mucositis.^9)^ In the 5-FU-induced COM rat model, Miyano et al. reported that continuous oral administration of HST (powder diet containing 1% HST) reduces the severity of oral ulcerative mucositis due to the enhanced healing of cutaneous wounds. Furthermore, Miyano and colleagues found that plasma concentrations of the ingredients of HST reached concentrations sufficient to exert the direct effects on oral mucositis [15].

Consistent with systemic administration, topical oral application of 100 mg/mL (10%) HST solution using an oral water flosser attenuates the oral mucositis score and the number of oral bacteria [14]. Similarly, topical oral application of 100 mg/mL (10%) HST solution by placing a cotton swab soaked with 20 μL of this solution on the labial fornix region alleviates pain-related behavior in an acetic acid-induced oral mucositis rat model [10]. These findings support the efficacy of topical oral application of HST in animal models of COM. However, to exert the inhibitory effect efficiently by topical oral application, the local concentration of active components and the contact time with COM are important. This is further supported by the present results that topical application of 5% TJ-14 solution (200 μL) twice per day exerted little effect on COM, although the same amount of HST extract (6 μg) was applied to the mucositis area higher frequently (twice per day) than the group treated with 12% HST oral ointment (50 μg) once per day. In this context, 12% HST or 20% TJ-14 oral ointment can fully meet these requirements. Furthermore, oral ointment is expected to have additional effects such as lubrication, moistening, and mechanical protection of the oral mucositis. Therefore, HST ointment may be a more effective treatment for COM than other oral applications of HST.

Previous studies have shown multiple pharmacological effects of HST and the underlying mechanisms in vivo and in vitro. HST exerts an anti-inflammatory effect by suppressing cyclooxygenase-2 (COX2) expression and prostaglandin E_2_ (PGE_2_) production [8, 9]. Among the ingredients of HST, [6]-shogaol and [6]-gingerol inhibit PGE_2_ metabolic activity without affecting COX2 expression, while baicalin, berberine, and wogonin inhibit COX2 expression [8]. HST removes free radicals and elicits an antimicrobial effect against Gram-negative bacteria [12–14]. [6]-shogaol and [6]-gingerol block voltage-gated Na^+^ channels (Na_v_1.8), which may contribute to the analgesic effect of HST [32]. In addition, recent studies demonstrate that HST enhances tissue repair through oral keratinocyte migration [15]. These multiple mechanisms may be responsible for the inhibitory effect of HST oral ointment on COM.

In conclusion, 12% HST oral ointment was successfully produced and demonstrated good pharmaceutical properties in terms of spreadability and stability. Moreover, topical application of 12% HST or 20% TJ-14 oral ointment to the oral mucosa was safe and elicited a therapeutic effect, rather than topical application of 5% TJ-14 solution, in an animal model of COM. We believe that the present results provide scientific evidence to promote clinical research of HST oral ointment for treatment of COM.

### Supplementary Information

Below is the link to the electronic supplementary material.

Supplementary file1 (DOCX 222 KB)
