# Correction: Preparation and pharmaceutical properties of Hangeshashinto oral ointment and its safety and efficacy in Syrian hamsters with 5-fluorouracil-induced oral mucositis

**DOI:** 10.1007/s11418-024-01806-1

**Published:** 2024-04-02

**Authors:** Takashi Ogihara, Masato Kagawa, Rintarou Yamanaka, Satoshi Imai, Kotaro Itohara, Daiki Hira, Shunsaku Nakagawa, Atsushi Yonezawa, Michiho Ito, Takayuki Nakagawa, Tomohiro Terada, Kazuo Matsubara

**Affiliations:** 1 Department of Clinical Pharmacology and Therapeutics, Kyoto University Hospital, 54 Shogoin-Kawahara-cho, Sakyo-ku, Kyoto, 606-8507 Japan; 2 Graduate School of Pharmaceutical Sciences, Kyoto University, 46-29 Yoshida-Shimoadachi-cho, Sakyo-ku, Kyoto, 606-8501 Japan; 3 Division of Pharmacognosy, Phytochemistry and Narcotics, Ministry of Health, National Institute of Health Sciences, Labour and Welfare, 3-25-26, Tonomachi, Kawasaki-ku, Kawasaki, Kanagawa 210-9501 Japan; 4 Department of Pharmacy, Wakayama Medical University, Wakayama, 641-8509 Japan

**Correction: Journal of Natural Medicines (2023) 77:53–63** 10.1007/s11418-022-01645-y

The article Preparation and pharmaceutical properties of Hangeshashinto oral ointment and its safety and efficacy in Syrian hamsters with 5-fluorouracil-induced oral mucositis, written by Takashi Ogihara, Masato Kagawa, Rintarou Yamanaka, Satoshi Imai, Kotaro Itohara, Daiki Hira, Shunsaku Nakagawa, Atsushi Yonezawa, Michiho Ito, Takayuki Nakagawa, Tomohiro Terada, Kazuo Matsubara, was originally published Online First without Open Access. After publication in volume 77, issue 1, pages 53–63 the author decided to opt for Open Choice and to make the article an Open Access publication. Therefore, the copyright of the article has been changed to © The Author(s) 2024 and the article is forthwith distributed under the terms of the Creative Commons Attribution 4.0 International License, which permits use, sharing, adaptation, distribution and reproduction in any medium or format, as long as you give appropriate credit to the original author(s) and the source, provide a link to the Creative Commons license, and indicate if changes were made. The images or other third party material in this article are included in the article’s Creative Commons licence, unless indicated otherwise in a credit line to the material. If material is not included in the article’s Creative Commons licence and your intended use is not permitted by statutory regulation or exceeds the permitted use, you will need to obtain permission directly from the copyright holder. To view a copy of this licence, visit http://creativecommons.org/licenses/by/4.0.

The original article was updated.

