# Supplementary material for: Preparation and pharmaceutical properties of Hangeshashinto oral ointment and its safety and efficacy in Syrian hamsters with 5-fluorouracil-induced oral mucositis
Source: J Nat Med. 2022 Aug 24;77(1):53–63. doi: 10.1007/s11418-022-01645-y (PMC11004029; doi:10.1007/s11418-022-01645-y)
Supplement: Supplementary file 1 — Supplementary file1 (DOCX 222 KB) [file 11418_2022_1645_MOESM1_ESM.docx]

**Supplementary Table 1** Grades for macroscopic and microscopic irritation of hamster cheek pouches in the oral mucosa irritation test

|  | Grade 0 | Grade 1 | Grade 2 | Grade 3 | Grade 4 |
| --- | --- | --- | --- | --- | --- |
| *Macroscopic reaction* | | | | | |
| Erythema | No erythema | Very slight erythema (barely perceptible) | Well-defined erythema | Moderate erythema | Severe erythema (beet-redness) or eschar formation preventing grading of erythema |
| *Microscopic reaction* | | | | | |
| Epithelium | Normal, intact | Cell degeneration or flattening | Metaplasia | Focal erosion | Generalized erosion |
| Leucocyte infiltration (number of leucocytes per high-power field) | Absent | Minimal (less than 25) | Mild (26–50) | Moderate (51–100) | Marked (more than 100) |
| Vascular congestion | Absent | Minimal | Mild | Moderate | Marked with disruption of vessels |
| Edema | Absent | Minimal | Mild | Moderate | Marked |


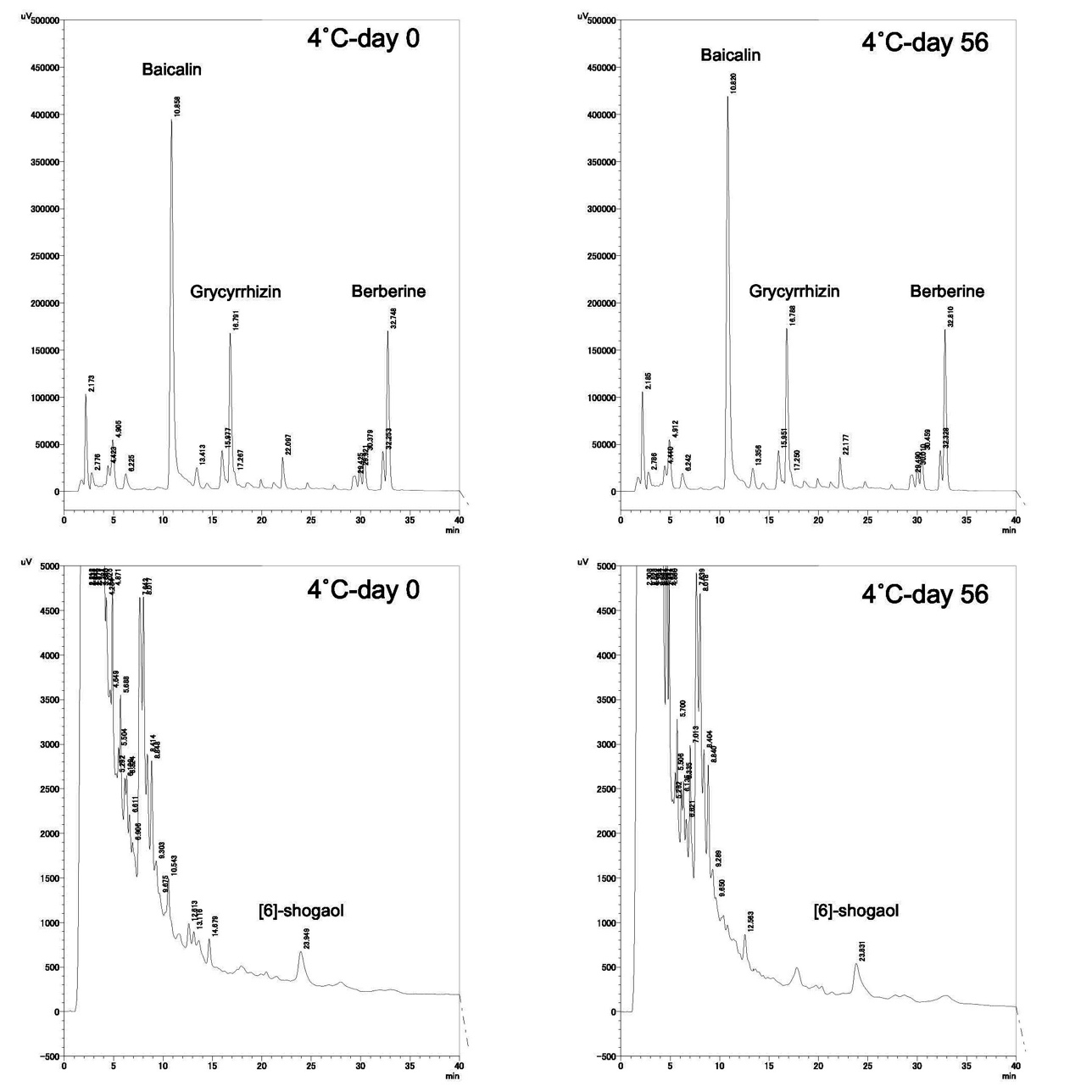


**Supplementary Figure 1** Representative HPLC chromatograms of the extracts from 12% HST oral ointments stored at 4˚C for 0 (before store) and 56 days in stability test. Upper and lower panels show the HPLC chromatograms according to the protocol of Okamura et al.^5)^ (baicalin, glycyrrhizin, and berberine) and Kano et al.^6)^ ([6]-shogaol), respectively.

**Supplementary Table 2** Macroscopic irritation scores of cheek pouches

| Group | Day 1 | | | | | |  | Day 2 | | | | | |  | | Day 3 | | | | | |  | Day 4 | | | | | |  | Day 5 | | | | | |
| --- | --- | --- | --- | --- | --- | --- | --- | --- | --- | --- | --- | --- | --- | --- | --- | --- | --- | --- | --- | --- | --- | --- | --- | --- | --- | --- | --- | --- | --- | --- | --- | --- | --- | --- | --- |
|  | 1st | | 2nd | | 3rd | |  | 1st | | 2nd | | 3rd | |  | | 1st | | 2nd | | 3rd | |  | 1st | | 2nd | | 3rd | |  | 1st | | 2nd | | 3rd | |
|  | L | R | L | R | L | R |  | L | R | L | R | L | R |  | L | | R | L | R | L | R |  | L | R | L | R | L | R |  | L | R | L | R | L | R |
| Sham | 0 | 0 | 0 | 0 | 0 | 0 |  | 0 | 0 | 0 | 0 | 0 | 0 |  | 0 | | 0 | 0 | 0 | 0 | 0 |  | 0 | 0 | 0 | 0 | 0 | 0 |  | 0 | 0 | 0 | 0 | 0 | 0 |
| TJ-14 ointment | 0 | 0 | 0 | 0 | 0 | 0 |  | 0 | 0 | 0 | 0 | 0 | 0 |  | 0 | | 0 | 0 | 0 | 0 | 0 |  | 0 | 0 | 0 | 0 | 0 | 0 |  | 0 | 0 | 0 | 0 | 0 | 0 |

| Group | Day 6 | | | | | |  | Day 7 | | | | | |  | | Day 8 | | | | | |  | Day 9 | | | | | |  | Day 10 | | | | | |
| --- | --- | --- | --- | --- | --- | --- | --- | --- | --- | --- | --- | --- | --- | --- | --- | --- | --- | --- | --- | --- | --- | --- | --- | --- | --- | --- | --- | --- | --- | --- | --- | --- | --- | --- | --- |
|  | 1st | | 2nd | | 3rd | |  | 1st | | 2nd | | 3rd | |  | | 1st | | 2nd | | 3rd | |  | 1st | | 2nd | | 3rd | |  | 1st | | 2nd | | 3rd | |
|  | L | R | L | R | L | R |  | L | R | L | R | L | R |  | L | | R | L | R | L | R |  | L | R | L | R | L | R |  | L | R | L | R | L | R |
| Sham | 0 | 0 | 0 | 0 | 0 | 0 |  | 0 | 0 | 0 | 0 | 0 | 0 |  | 0 | | 0 | 0 | 0 | 0 | 0 |  | 0 | 0 | 0 | 0 | 0 | 0 |  | 0 | 0 | 0 | 0 | 0 | 0 |
| TJ-14 ointment | 0 | 0 | 0 | 0 | 0 | 0 |  | 0 | 0 | 0 | 0 | 0 | 0 |  | 0 | | 0 | 0 | 0 | 0 | 0 |  | 0 | 0 | 0 | 0 | 0 | 0 |  | 0 | 0 | 0 | 0 | 0 | 0 |

| Group | Day 11 | | | | | |  | Day 12 | | | | | |  | | Day 13 | | | | | |  | Day 14 | | | | | |  | Day 15 | |
| --- | --- | --- | --- | --- | --- | --- | --- | --- | --- | --- | --- | --- | --- | --- | --- | --- | --- | --- | --- | --- | --- | --- | --- | --- | --- | --- | --- | --- | --- | --- | --- |
|  | 1st | | 2nd | | 3rd | |  | 1st | | 2nd | | 3rd | |  | | 1st | | 2nd | | 3rd | |  | 1st | | 2nd | | 3rd | |  |  |  |
|  | L | R | L | R | L | R |  | L | R | L | R | L | R |  | L | | R | L | R | L | R |  | L | R | L | R | L | R |  | L | R |
| Sham | 0 | 0 | 0 | 0 | 0 | 0 |  | 0 | 0 | 0 | 0 | 0 | 0 |  | 0 | | 0 | 0 | 0 | 0 | 0 |  | 0 | 0 | 0 | 0 | 0 | 0 |  | 0 | 0 |
| TJ-14 ointment | 0 | 0 | 0 | 0 | 0 | 0 |  | 0 | 0 | 0 | 0 | 0 | 0 |  | 0 | | 0 | 0 | 0 | 0 | 0 |  | 0 | 0 | 0 | 0 | 0 | 0 |  | 0 | 0 |

L (left cheek pouch), not treated; R (right cheek pouch), treated (sham or TJ-14 ointment).

Score 0, no erythema; score 1: very slight erythema; score 2, well-defined erythema; score 3, moderate erythema; and score 4: severe erythema (beet-redness) or eschar formation preventing grading of erythema.

Scores were evaluated before each administration (first, second, and third) on each day during the administration period (from day 1 to day 14). Each value represents mean. *n* = 3.

**Supplementary Table 3** Microscopic irritation scores of cheek pouches

| Finding | Sham | | | |  | TJ-14 ointment | | | |
| --- | --- | --- | --- | --- | --- | --- | --- | --- | --- |
|  | Distal | Middle | Proximal | All |  | Distal | Middle | Proximal | All |
| Epithelium | 0 | 0 | 0 | 0 |  | 0 | 0 | 0 | 0 |
| Leucocyte infiltration | 0 | 0 | 0 | 0 |  | 0 | 0 | 0 | 0 |
| Vascular congestion | 0 | 0 | 0 | 0 |  | 0 | 0 | 0 | 0 |
| Edema | 0 | 0 | 0 | 0 |  | 0 | 0 | 0 | 0 |

Microscopic irritation scores of the oral mucosa in the distal, middle, and proximal sites of cheek pouches were evaluated on day 15.

Epithelium: score 0, normal and intact; score 1, cell degeneration or flattening; score 2, metaplasia; score 3, focal erosion; and score 4, generalized erosion.

Leucocyte infiltration: score 0, absent; score 1, minimal (<25 per high power field); score 2, mild (26–50 per high power field); score 3, moderate (51–100 per high power field); and score 4, marked (>100 per high power field).

Vascular congestion: score 0, absent; score 1, minimal; score 2, mild; score 3, moderate; and score 4, marked with disruption of vessels.

Edema: score 0, absent; score 1, minimal; score 2, mild; score 3, moderate; and score 4, marked.

Each value represents mean. *n* = 3.
